# Use of a novel smart heating sleeping bag to improve wearers’ local thermal comfort in the feet

**DOI:** 10.1038/srep19326

**Published:** 2016-01-13

**Authors:** W. F. Song, C. J. Zhang, D. D. Lai, F. M. Wang, K. Kuklane

**Affiliations:** 1 Laboratory for Clothing Physiology and Ergonomics (LCPE), The National Engineering Laboratory for Modern Silk, Soochow University, 199 Ren’ai Road, Suzhou, 215123 China; 2 College of Textile and Clothing Engineering, Soochow University, 178 Ganjiang East Road, Suzhou 215021, China; 3 Thermal Environment Laboratory, Department of Design Sciences, Lund University, Lund 22100, Sweden

## Abstract

Previous studies have revealed that wearers had low skin temperatures and cold and pain sensations in the feet, when using sleeping bags under defined comfort and limit temperatures. To improve wearers’ local thermal comfort in the feet, a novel heating sleeping bag (i.e., MAR_HT_) was developed by embedding two heating pads into the traditional sleeping bag (i.e., MAR_CON_) in this region. Seven female and seven male volunteers underwent two tests on different days. Each test lasted for three hours and was performed in a climate chamber with a setting temperature deduced from EN 13537 (2012) (for females: comfort temperature of −0.4 °C, and for males: the limit temperature of −6.4 °C). MAR_HT_ was found to be effective in maintaining the toe and feet temperatures within the thermoneutral range for both sex groups compared to the linearly decreased temperatures in MAR_CON_ during the 3-hour exposure. In addition, wearing MAR_HT_ elevated the toe blood flow significantly for most females and all males. Thermal and comfort sensations showed a large improvement in feet and a small to moderate improvement in the whole body for both sex groups in MAR_HT_. It was concluded that MAR_HT_ is effective in improving local thermal comfort in the feet.

A sleeping bag is an essential textile product that is aimed at protecting people in cool or cold outdoor environments. They are widely used in field training, rescue and relief work and are also becoming common equipment in travelling and recreation for ordinary people^1^. Traditional sleeping bags are generally composed of an outer layer, an inner layer and the filling. Sleeping bags are often required to be marked with four labelled temperatures according to the EN 13537 (2012) standard^2^: maximum temperature, comfort temperature, limit temperature, and the extreme temperature. Of all these four labelled temperatures, comfort temperature and limit temperature are the two most important temperatures for users. Comfort temperature indicates the lower threshold temperature at which a standard woman could sleep for eight hours without cold feeling in a relaxed posture (i.e., lying on their back) under the standard condition. The limit temperature refers to the lower threshold temperature for a standard man sleeping in a cured up body posture for eight hours without feeling cold under the standard condition. Previous studies have shown that the comfort temperature and limit temperature were sometimes questioned by users and scientists^3^. This is because some manufacturers defined these two temperatures based on qualitative methods, such as customers’ feedback and assessments from bag’s thickness, weight and loftiness^4^.

Many models have been developed to compute the operating temperatures of the sleeping bag. For example, the EN 13537 (2012) model^2^, the KSU model^5^, Belding’s model^6^, Goldman’s model^7^, Holand’s model^8^, den Hartog’s model^9^, and the IREQ (Required Clothing Insulation) model^10^. However, all these models calculate the global comfort and limit temperatures, i.e., these models only considered the whole body thermal comfort^5^. It seems that both the comfort temperature and the limit temperature defined by the models failed to take care of local thermal comfort in the feet^8^. Lin *et al.*^3^ investigated the physiological and psychological responses of females and males in different sleeping bags under both the EN 13537 (2012) and the IREQ model defined temperatures. Although the mean skin temperature was within its thermal neutral range, almost all subjects had continuously dropping toe temperatures throughout the two-hour exposure. The temperature dropping in the toes has already caused a great sensation of cold and pain. It was thus concluded that such defined temperatures were unable to allow users to have an eight-hour comfortable sleep^3^. Huang^11^ also observed continuously decreasing toe temperatures on both females and males during the eight-hour wear trials under environmental temperature settings defined by five models (i.e., the EN 13537 model, the KSU model, Belding’s model, Goldman’s model and den Hartog’s model). These studies indicated that the operating temperatures defined for sleeping bags provided insufficient protection at the body extremities.

The extremities of human body are more sensitive to cold exposure compared to other body regions because of the large specific surface area and the low local metabolic heat production in these regions^12^. Skin temperature dropping normally appears in those regions initially, and results in the first sign of cold and pain sensations in those local regions^11^,^12^. Besides the induced local thermal discomfort, the whole body thermal comfort was also found to be strongly related with the extremity temperatures^11^,^12^. It is reasonable to speculate that heating the extremity, e.g., the feet region, could prevent the local skin temperature dropping, and thereby improve local thermal comfort of the human body.

In this study, a novel smart heating sleeping bag was developed by embedding two electrical heating pads into traditional sleeping bags at the feet region. It is anticipated that the smart heating sleeping bag is useful in such outdoor settings as camping sites and recreational facilities. The physiological responses and perceptual responses when using the traditional sleeping bag and newly developed smart heating bag were examined and compared. It was hypothesized that local thermal comfort could be improved by using the smart heating sleeping bag in cold outdoor environments.

## Methods

### Subjects

Seven female and seven male subjects voluntarily participated in this study. They were physically healthy and had no smoking habit. They also had limited experience in using sleeping bags. The average age, height, weight, body surface area and the body mass index of the females are 24.0 ± 1.4 yr., 160.9 ± 2.8 cm, 52.1 ± 4.9 kg, 1.53 ± 0.07 m^2 ^and 20.1 ± 1.6 kg/m^2^, respectively, and those of males are 24.9 ± 1.7 yr., 172.8 ± 2.6 cm, 62.0 ± 3.5 kg, 1.74 ± 0.06 m^2 ^and 20.8 ± 0.9 kg/m^2^, respectively. They were asked not to drink alcohol and do intensive exercises at least twenty-four hours before the test. Also, they were not allowed to drink tea or coffee at least two hours before each trial. This study was approved by the ethical committee of Soochow University and was strictly performed in agreement with legal requirements and international norms (Declaration of Helsinki, 1964). The research methods are in accordance with the approved guidelines. Subjects were informed about the purpose, the procedure and potential risks associated with the experiment. Each participant signed a written consent form prior to participation.

### Sleeping bag

A mummy-shaped sleeping bag (code: MAR) was randomly selected from the market. Based on this sleeping bag, a novel smart sleeping bag was developed by embedding two electrical heating pads into MAR at the feet region. The heating pad was engineered by sandwiching a 2.25 m winding carbon heating wire (total electric resistance: 322 Ω) between two high-density polyester fabric layers (length × width × thickness: 38 cm × 38 cm × 0.159 cm). The total weight of two heating pads was 464 g. The heating pads were connected to a power supply and the heating power could be adjusted from 0 to 45 W. In this study, two levels of heating power was selected, 0 W (i.e., heating was turned off) and 20 W (i.e., close to medium heating power), corresponding to two testing scenarios written as MAR_CON_ and MAR_HT_, respectively. MAR_CON_ represents the non-heating traditional sleeping bag, and MAR_HT_ is the smart heating sleeping bag to be examined in this study. The selection of a constant heating power (i.e., 20 W) was based on previous findings of our pilot study^27^, in which the heating power was stepwise adjusted from 0 to 45 W with 5 W increments. Both male and female subjects (i.e., 4 males and 4 females) rated thermoneutral when the power was set to 20 W during three-hour sleep time under the same environmental conditions as those used in this study. The structure, composition, and labelled comfort (*T*_*com-l*_) and limit temperatures (*T*_*lim-l*_) of the two sleeping bags are listed in Table 1. The comfort (*T*_*com-c*_) and limit temperatures (*T*_*lim-c*_) were translated from their perspective thermal insulation values according to the EN 13537 (2012). The thermal insulation values were determined by a ‘Newton’ thermal manikin (Measurement Technology Northwest, Seattle, USA), strictly followed the EN 13537^2^. The detailed manikin test procedure was described as follows: the sleeping bag was taken out from the package, and then conditioned for at least 12 hours before each manikin test to eliminate the packing effect on thermal insulation. The manikin was dressed with long cotton underwear (including a sweater and long trousers) with a thermal insulation of 0.049 °C·m^2^/W, knee-length socks and the face mask. The manikin was then placed in the sleeping bag, and the zipper and the hood were tightly closed. The whole manikin system was placed on an inflated mattress with a thickness of 40 mm (Therm-a-Rest, Cascade designs Inc., Seattle, USA), underneath which there was a 12 mm thick wooden board. The thermal insulation of the sleeping bags was calculated by the serial method (see the calculation part in this section)^2^, which was 6.8 clo and 7.4 clo (1 clo = 0.155 m^2^·K/W), respectively. Obviously, both the *T*_*com-c*_and *T*_*lim-c*_ of MAR_CON_ are higher than its *T*_*com-l*_and *T*_*lim-l*_, respectively. Moreover, MAR_HT_ lowered both *T*_*com-c*_ and *T*_*lim-c*_ compared to MAR_CON_.

### Test conditions

For the manikin tests, the ambient temperature was set to −2 ± 0.5 °C, the relative humidity was set to 80 ± 5% and the air velocity was controlled at 0.5 ± 0.1 m/s. All human trial tests were conducted in a climate chamber with different setting temperatures for females and males. For females, they were exposed to the air temperature of −0.4 °C (i.e., the comfort temperature of MAR_CON_) in both MAR_CON_ and MAR_HT_, and for males, they were exposed to −6.4 °C (i.e., the limit temperature) in the two sleeping bags (i.e., MAR_CON_ and MAR_HT_). The air temperature fluctuations were controlled within ± 0.5 °C for both genders. The relative humidity and air velocity in the chamber were set as 80 ± 5% and 0.5 ± 0.1 m/s, respectively.

### Test protocol

Each participant was asked to sleep in the smart heating bag (MAR_HT_) and the traditional sleeping bag (MAR_CON_) in a randomized and counter-balanced order. A total of 28 tests were performed (i.e., 14 participants times 2 tests). Each test was conducted at the night time and the same time of a night with an interval of at least 48 hours in between to eliminate the circadian variation impact. The participants were blinded to the sleeping bags to avoid prejudice. They were dressed with the same underwear and knee-length socks that were used in the manikin tests.

Upon arrival for the tests for the sleeping bag, the participants were asked to rest on a chair for 15–30 min at a room temperature of 20.0 °C, and then instrumented. After the preparation, the participants entered the climatic chamber (where the test condition was set based on the assigned test scenario) and laid into the bag randomly assigned in a flat lying posture. This transition period (from the outside room to the chamber) normally took about 5 min. The sleeping bag was placed on an inflated mattress supported by a wooden table. The participants were asked to stay in the sleeping bags for a maximum of 3 hours. The test was terminated when any of the following criteria were met: i) the toe temperature dropped to 12.0 °C; ii) the participant refused to continue; and iii) the 180 min testing was achieved. After the test, the participants came out of the chamber and they were asked to take off the clothing and the equipment.

Metabolic rate was measured continuously for 5 min with a cardiopulmonary tester at the 20^th^ min of the test (MetaMax®3B, Cortex Biophysik GmbH, Leipzig, Germany). Skin temperatures on eleven sites, namely, forehead, upper arm, forearm, chest, specula, hand, ring finger, thigh, calf, left foot and the 4^th^ toe, were measured by temperature sensors (MSR®145B4, MSR Electronic GmbH, Seuzach, Switzerland). Blood flow of the 4^th^ toe was measured using a Doppler-type laser flow meter (ALF 21R, Advance Co., Ltd, Tokyo, Japan). Heart rate was obtained via a Polar^®^ chest strap and a heart rate watch (Polar Electro Oy, Kempele, Finland). The skin temperatures, the skin blood flow and the heart rate were logged at an interval of 30 s throughout the test.

**Figure 1:**
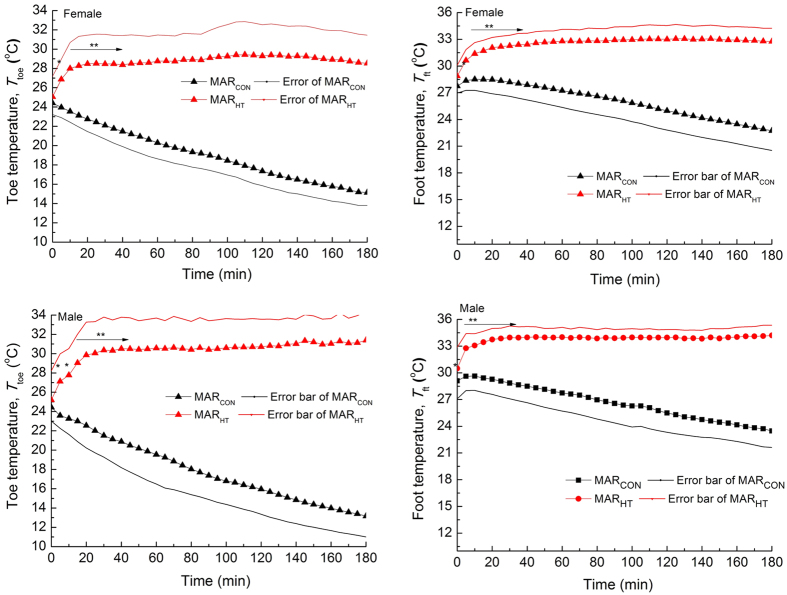
The evolution curves of the 4^th^ toe and the left foot temperatures for females and males in MAR_CON_ and MAR_HT_.

Perceptual sensations of the participants in the hands and feet as well as the whole body were rated 15 min before the test, in the beginning of the test, at the 20^th^ and the 180^th^ min of the test (Fig. 1). Each rating of the perceptual sensations was explained to the participants in detail prior to the trial. Thermal sensation (TS) of “−4” corresponds to very cold, “−3” cold, “−2” cool, “−1” slightly cool, “0” neutral, “1” slightly warm, “2” warm, “3” hot, and “4” very hot. Comfort sensation (CS) of “−3” corresponds to very uncomfortable, “−2” uncomfortable, “−1” slightly uncomfortable, and “0” neutral. Skin wetness sensation (WS) of “−3” corresponds to very dry, “−2” dry, “−1” slightly dry, “0” neutral, “1” slightly wet, “2” wet and “3” very wet. The perceptual ratings were verbally reported by the participants at the time points mentioned above.

### Calculations

The thermal insulation (*I*_t_, in clo) of the sleeping bag was computed by the serial method defined by EN 13537, expressed as equation (1).


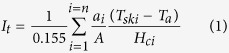


where, *A* is the manikin surface area (i.e., 1.697 m^2^); *n* is the segment number, *n* = 32; *a*_*i*_ is the surface area of the segment *i*, m^2^; *H*_ci_ is the heat flux of the *i* segment, W/m^2^; *T*_ski_ is the skin temperature of the segment *i*, °C; *T*_a_ is the ambient air temperature, °C.

The mean skin temperature (*T*_sk_) was calculated using the Gagge and Nishi’s equation (Gagge and Gonzalez, 2011), expressed as equation (2).


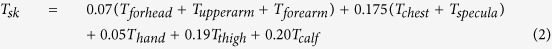


### Statistical analysis

Data were presented as mean ± standard deviation (SD). A two-way repeated measures ANOVA [Test scenarios (MAR_HT_ vs. MAR_CON_)] × Time [(i.e., the 0^th^, 10^th^, 20^th^, 40^th^, 60^th^, 80^th^, 100^th^, 120^th^, 140^th^, 160^th^, and the 180^th^ min)] was performed using SPSSv.20 (IBM Inc., Armonk, NY) to assess the differences in the physiological responses between the two test conditions, time effect and the interaction effect between conditions and the time. When the Mauchly’s Test of Sphericity was violated, the Greenhouse-Geisser correction was employed as statistical significance. When the repeated measures showed a significant effect, a paired sample t-test for was conducted at each time point. The significance levels were *p* < 0.05 (marked * on the graphs) and < 0.01 (**) in all tests. The bias-corrected hedge’s effect sizes (standardized mean difference) were calculated for evaluating the differences in perceptual responses at the two time points (the 20^th^ min and the 180^th^ min) between the two conditions and also between the two points (Hedges *et al.*, 1985). The effect sizes (EFSs) were obtained using the Review Manager V.5.2.0 software, involving the input parameters of mean outcome, the standard deviation and the sample size. The magnitude of EFS was interpreted as: 0–0.19 = negligible effect, 0.20–0.49 = small effect, 0.50–0.79 = moderate effect and >0.8 = large effect. Negative effects of MAR_HT_ and time were displayed with a minus sign.

## Results

### Metabolic rate

All subjects finished the 180 min test. Metabolic rate was not significantly different between MAR_CON_ and MAR_HT_ for both females (i.e., 1.1 ± 0.1 METs in MAR_CON_ and 1.2 ± 0.2 METs in MAR_HT_, *p* > 0.05) and males (i.e., 1.1 ± 0.2 METs in MAR_CON_ and 1.1 ± 0.2 METs in MAR_HT_, *p* > 0.05).

### The 4^th^ toe and foot temperatures

Figure 1 presented the evolution curves of the *T*_toe_ and *T*_ft_ curves for females and males in MAR_CON_ and MAR_HT_. Significantly higher *T*_toe_ and *T*_ft_ were detected for both females and males in MAR_HT_ compared to MAR_CON_ from the 5^th^ min to the end of the test (*p* < 0.01). Linearly dropping *T*_toe_ and *T*_ft_ were detected for both females and males in MAR_CON_ (main time effect, *p* < 0.01), while *T*_toe_ and *T*_ft_ significantly increased during the initial 20 min (main time effect, *p* < 0.01) and then remained stable in MAR_HT_ throughout the remaining test duration. At the end of the test, *T*_toe_ and *T*_ft_ of females in MAR_CON_ sank to 15.1 °C and 22.7 °C, respectively, and for males to 13.2 °C and 23.5 °C, respectively. Comparing the average *T*_toe_ and *T*_ft_ values in the stable stage (time range from the 20^th^ min to the 180^th^ min) for the two sex groups in MAR_HT_, *T*_toe_ was higher for males (i.e., 30.7 ± 2.8 °C) than that for females (i.e., 28.9 ± 2.7 °C), though not significant (*p* > 0.1), while *T*_ft_ was significantly higher on females (33.9 ± 1.0 °C) than that on males (i.e., 32.8 ± 1.4 °C ) (*p* < 0.05).

### The 4^th^ toe blood flow

**Figure 2:**
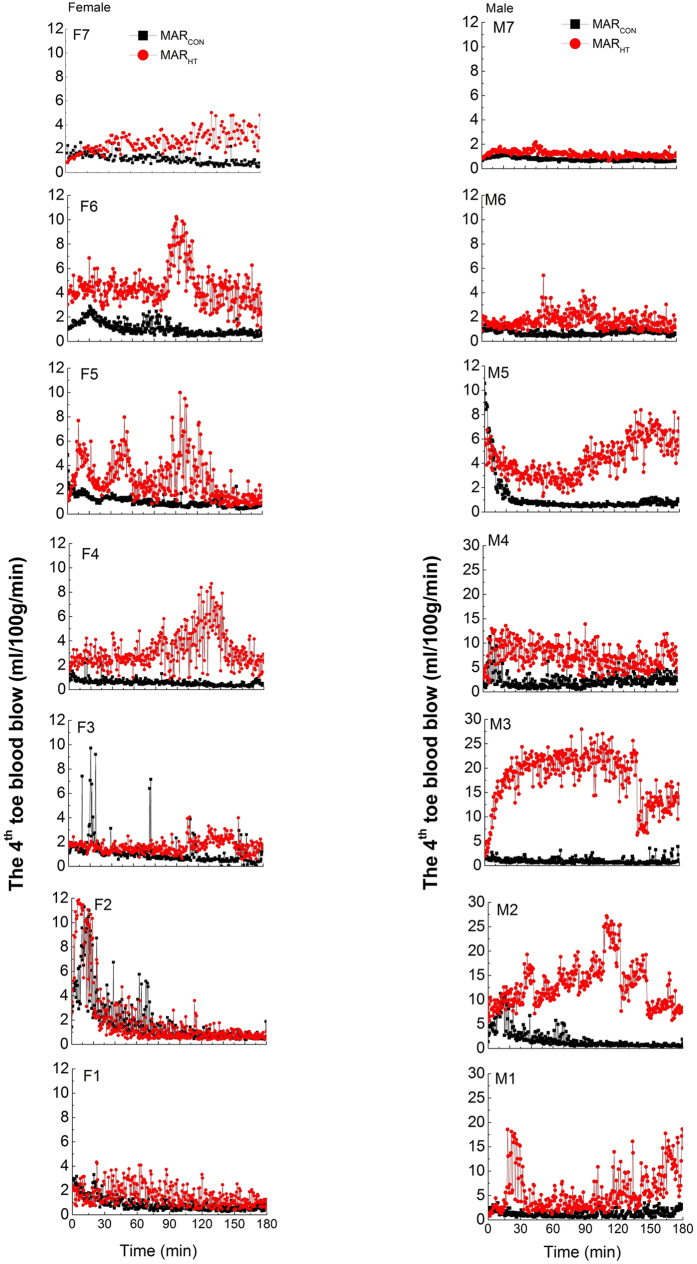
The evolution curves of the 4^th^ toe blood flow for females and males. F1 to F7 means females numbered from 1 to 7, M1 to M7 means males numbered from 1 to 7.

The evolution curves of the 4^th^ toe blood flow (SkBF) for all the fourteen subjects in MAR_CON_ and MAR_HT_ were listed in Fig. 2. The average values of SkBF from the 20^th^ min to the end of test were calculated for all the subjects (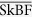). SkBF decreased to near zero values with the exposure time for all the subjects in MAR_CON_. While SkBF values fluctuated at relatively higher values at different levels for the subjects (except for F1) in MAR_HT_ (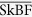 ranging from 1.2 to 4.6 ml/100g/min and 1.2 to 18.8 ml/100g/min for females and males, respectively) compared to those in MAR_CON_ (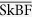 ranging from 0.5 to 1.3 ml/100g/min and 0.6 to 2.1 ml/100g/min for females and males, respectively). It was also noted that the 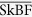 on five out of seven males (4.8 to 18.8 ml/100g/min) in MAR_HT_ exhibited higher values compared to all females (1.2 to 4.6 ml/100g/min) in MAR_HT_.

### Mean skin temperature and mean heart rate

**Figure 3:**
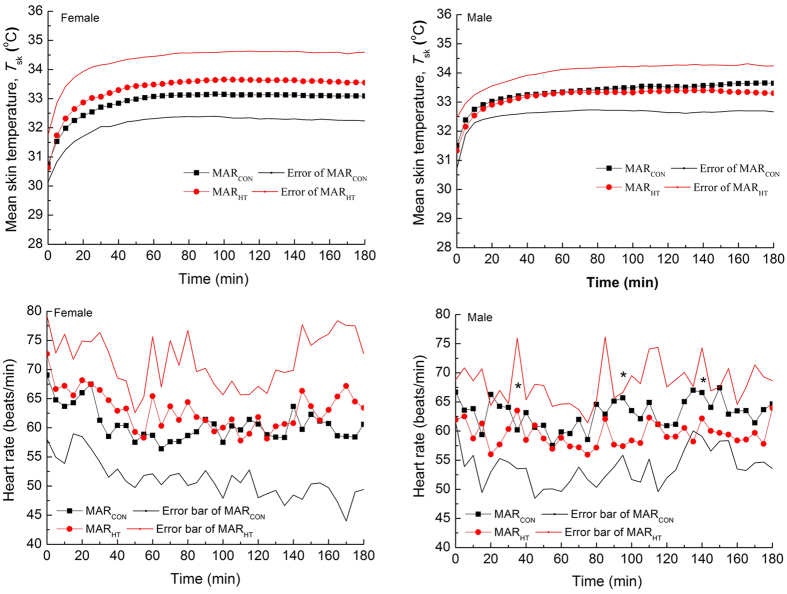
The evolution curves of the mean skin temperature and heart rate for females and males in MAR_CON_ and MAR_HT_.

The mean skin temperature (*T*_sk_) in MAR_CON_ and MAR_HT_ significantly increased during the first 20 min (time main effect, *p* < 0.01) and then were kept stable throughout the whole test (Fig. 3). No significant difference was observed in *T*_sk_ between MAR_CON_ and MAR_HT_ for both females and males (*p* > 0.1). The average *T*_sk_ data in the stable stage for females (i.e., 33.0 ± 0.8 °C in MAR_CON_ and 33.2 ± 0.9 °C in MAR_HT_) were close to those of males (i.e., 33.2 ± 0.6 °C in MAR_CON_ and 33.3 ± 0.8 °C in MAR_HT_) (see Fig. 3, *p* > 0.1).

Heart rate fluctuated with the testing time and no time main effect was observed (*p* > 0.05). No significant difference in heart rate was detected for females in MAR_HT_ compared to MAR_CON_ (*p* > 0.1). Heart rate was with similar average values for females in MAR_CON_ (i.e., 60.5 ± 7.8 beats/min) and MAR_HT_ (i.e., 63.1 ± 7.2 beats/min). However, significantly lower heart rate was observed on males in MAR_HT_ compared to those on males in MAR_CON_ at the 25^th^ min, the 95^th^ min and the 150^th^ min of the test (*p* < 0.05) (Fig. 3). Besides, the average heart rate on males in MAR_HT_ (i.e., 59.0 ± 7.3 beats/min) was higher compared to that on male subjects in MAR_CON_ (i.e., 63.0 ± 7.6 beats/min) (*p* = 0.052) and the difference was approaching statistical significance. The average heart rate values between the two genders showed no significant difference in neither MAR_CON_ (*p* > 0.1) or MAR_HT_ (*p* > 0.1).

### Perceptual responses

As listed in Table 2 and Table 3, all subjects stayed in a thermal neutral state (TS, CS and WS = 0) before and at the start of the cold exposure, as well as at the 20^th^ min of the exposure except for TS of females in MAR_CON_. Perceptual responses deviated from zero in different levels at the end of cold exposure.

Comparing the perceptual data at the end of the test with those at the start, TS and CS in the feet are subjected to the most obvious declines for both females and males in MAR_CON_ (negative large EFSs). The evolution of the TS and CS in the hand for the two sex groups in MAR_CON_ was not apparent (negative small EFSs). TS alterations in the whole body are moderate for females (negative moderate EFSs) and small for males in MAR_CON_ (negative small EFSs). CS alterations in the whole body are small for both genders in MAR_CON_ (negative small EFSs). Wetness sensation showed no change in the feet or in the whole body, and was small in the hand for the two genders in MAR_CON_.

Concerning the perceptual data in the feet between MAR_CON_ and MAR_HT_ at the 180^th^ min of the exposure, cool to cold sensations were detected for both females and males in MAR_CON_, while slightly cool and slightly warm sensations were observed for them in MAR_HT_, respectively (MAR_HT_ vs. MAR_CON_: large EFSs). Slightly uncomfortable to uncomfortable ratings were nominated by both genders in MAR_CON,_ while neutral comfort sensation was rated by them in MAR_HT_ (MAR_HT_ vs. MAR_CON_: large EFSs). No skin wetness sensation was perceived by the participants for all the test conditions.

With respect to perceptual data in the hand, slightly cool sensations were detected for both females and males in MAR_CON_, while slightly warm and neutral sensations were observed for females and males in MAR_HT_, respectively (MAR_HT_ vs. MAR_CON_: moderate EFSs). Slightly uncomfortable to uncomfortable ratings were nominated by both genders in MAR_CON,_ while neutral comfort sensation was rated by them in MAR_HT_ (MAR_HT_ vs. MAR_CON_: large EFSs). Skin wetness sensations of slightly wet were perceived by the two sex groups in both MAR_CON_ and MAR_HT_ (MAR_HT_ vs. MAR_CON_: negligible EFSs).

As for the whole body, slightly cool sensation was perceived by both sex groups in MAR_CON_, while slightly warm sensation and the neutral sensation were detected in MAR_HT_ (MAR_HT_ vs. MAR_CON_: moderate EFSs for females and small EFSs for males), respectively. Slightly uncomfortable to uncomfortable ratings were nominated by both genders in MAR_CON,_ whereas neutral comfort sensations were rated in MAR_HT_ (MAR_HT_ vs. MAR_CON_: moderate and small EFSs for females and males, respectively). No wetness sensation was detected.

Perceptual sensations of females and males in MAR_CON_ and MAR_HT_ showed approximate results except for TS in the feet in MAR_HT_. Slightly cool sensation was noted in the feet on females, while slightly warm sensation was reported on males.

## Discussion

Sleeping bags serve as an important protective textile product for human body while sleeping under cool or cold outdoor environments. A sleeping bag should allow the wearers to sleep for four to eight hours without feeling cold under the bags’ operating temperatures^13^. Even though the total duration of each trial in this study was 3 hours, the linearly decreasing toe (*T*_toe_) and foot temperatures (*T*_ft_) (see Fig. 1) could cause pronounced cold sensations in the feet for both genders^14^. Thus, our results have reconfirmed that the local region of the human body was inadequately protected while using the studied sleeping bag under the environmental temperatures defined by EN 13537 (2012). Lin *et al.*^3^ detected that *T*_toe_ of females in a traditional sleeping bag decreased to 21.1 °C at the 120^th^ min while exposed to EN 13537 defined comfort temperature (i.e., 3.8 °C). In this study, *T*_toe_ of females decreased to 21.3 °C at the 120^th^ min. However, Lin *et al.*^3^ observed no declination of *T*_toe_ for males in MAR_CON_ under EN 13537 defined limit temperature (i.e., 11.2 °C and 2.1 °C). The discrepancy might be due to the lower exposure temperature (i.e., −6.8 °C) adopted in this study, reconfirming that the human body extremity was more affected by environmental temperatures compared to other body parts.

Further, it is evident that the MAR_HT_ could well maintain *T*_toe_ and *T*_ft_ of both females (i.e., *T*_toe_: 28.9 ± 2.7 °C and *T*_ft_: 33.9 ± 1.0 °C) and males (i.e., *T*_toe_: 30.7 ± 2.8 °C and *T*_ft_: 32.8 ± 1.4 °C) in a thermoneutral range (i.e., 25.0–34.0 °C)^13^. The heating pads had two positive effects in preventing the toe and foot temperature dropping, i.e., creating a warm microclimate and raising the local apparent thermal insulation at the feet region due to the added heating layer. As no investigation was conducted on smart heating sleeping bags, we could only retrieve literature regarding the effect of heated footwear on the local skin temperature. van Somerenet *et al.*^15^ narrated that heating the feet would keep the feet temperature the same as the rest of the body and suggested that the feet play a significant role in the whole body thermoregulatory response to cold. Isik^16^ also found heating the feet could provide stable skin temperature in the region, but it exerted no effect on the other parts of the body.

Supplying heat to the feet region could promote the local skin blood flow (i.e., SkBF). House *et al.*^17^ compared the effects of the heated and non-heated socks on the feet and discovered that the skin blood flow at the toes in heated socks was much greater than that in non-heated socks. In this study, greater skin blood flow at the 4^th^ toe in MAR_HT_ than that in MAR_CON_ was also observed on most subjects. The mechanism behind the toe blood flow was consistently pinpointed by researchers to depend on the action modes of arteriovenous anastomoses (AVAs) (i.e., small blood vessels that interconnect small veins with small arteries), which controlled 90% of the blood flow in the local regions^18^, and was abundantly presented in the extremities^18^,^19^,^20^. The action modes of AVAs relied on the ambient temperature of the extremities^18^,^19^, which expressed vasoconstriction (to reduce heat loss) and vasodilatation (to increase heat loss) modes under low and high temperatures, respectively. It is thus reasonable to speculate that the close to zero SkBF values for subjects in MAR_CON_ was induced by the vasoconstriction of AVAs in the cold microenvironment between foot and MAR_CON_, while the promoted SkBF values for the subjects in MAR_HT_ was attribute to vasodilatation of AVAs in the warm microenvironment created by the MAR_HT_. Individual difference exists expressed by the unaffected SkBF evolutions of one female and different levels of SkBF promotions for the rest subjects in the two sleeping bags.

The relationship between SkBF and *T*_toe_ when supplying feet with exogenous heat was investigated by some researchers^21^,^22^. Song^21^ conducted a study by having subjects touching heating floor with different surface temperatures, and discovered that the blood flow rate in the toe at the dorsal part increased with the increasing toe temperature ranging from 20.0 to 34.0 °C (sharp increment occurs at from 30.0 to 34.0 °C). Allwood *et al.*^22^ applied increased local temperatures at feet and discovered that SkBF increased gradually from temperature of 15 to 29 °C and then remarkably from 29 to 32 °C. The SkBF of most males in MAR_HT_ was higher compared to that of all females, which might be obtained from the higher toe temperatures (i.e., 30.7 ± 2.5 °C) on males than on females (28.9 ± 2.7 °C) or from the AVAs difference between the two genders.

Heating the feet region exerted no effect on the whole body temperature. Similar findings were also observed by Isik^16^, who detected that heating the feet played no effect on the body temperature at other regions. All the observed *T*_sk_ values were laid in the thermal neutral range of 32.0–34.0 °C 19. Heart rate fluctuated with the testing time in the range of 60 to 80 beats/min (bpm). Our results reconfirmed that the EN 13537 (2012) defined temperatures only considered the whole body thermal balance rather than sleeping thermal comfort. Significantly lower heart rate was detected on males in MAR_HT_ compared to MAR_CON_ at some points during the 3-hour exposure. In addition, MAR_HT_ showed a much lower average heart rate during the 3-hour exposure compared to MAR_CON_ and approached the statistical significance indicated by *p* = 0.052. Some researchers^23^,^24^ noted that there exists a connection between blood flow fluctuations through AVAs in the fingers and toes, and spontaneous heart rate as well as the blood pressure variability. In this study, the significant heart rate difference observed on males might be caused by the high toe blood flow in MAR_HT_. Future studies should be conducted to ascertain the relationship between these two variables, however.

Given the physiological responses in the feet, it is not surprising to find that the perceptual responses (TS and CS) of participants in MAR_CON_ deteriorated with the exposure time. Local perceptual responses play an important role in affecting the whole body sensations^11^,^25^. Arens *et al.*^26^ opined that cold feet would disrupt the whole body thermal comfort even if the rest of the body was properly dressed. Huang^6^ detected that TS in the feet is highly correlated to that in the whole body (the Spearman correlation coefficient of 0.8). Though no aggravated global physiological responses (i.e., the mean skin temperature and the heart rate), it is still inevitably to observe the disruption of the whole body perceptual responses (TS and CS) in MAR_CON_, which might be aroused by the worsened local thermal responses in the feet. Our findings further demonstrate that the EN 13537 (2012) failed to protect the local body region. Therefore, it is highly necessary to revise the EN 13537 (2012) to address the issue on the protection of local body areas, e.g. feet.

In MAR_HT_, a large improvement of perceptual responses (TS and CS) in the feet was observed, and the resultant small to moderate improvement in the whole body sensation was thus detected. Slight wetness sensation was detected in the hands regardless of the sleeping bag types, which might be explained from the aspect of the hand warming due to the close contact between hands and the torso^3^. The perceptual responses (TS, CS and WS) in MAR_HT_ have demonstrated the advantage of the newly developed smart heating sleeping bag over traditional sleeping bags.

Perceptual responses of females and males in MAR_HT_ and MAR_CON_ were consistent except for TS in the feet (slightly warm and cool sensations for males and females, respectively). This was in accordance with and also might be explained from the difference of physiological responses in feet for the two sex groups. Females exhibited significantly lower foot temperatures and blood flow than males.

Finally, there are generally two potential approaches to protect the human extremities such as the feet when sleeping outdoors: selecting a high insulation bag and using it at higher temperatures than the EN 13537 (2012) defined operating temperatures or supplying heating to the feet regions. Although the first method seems effective, high insulation sleeping bags are bulky and costly. Hence, supplying heat to the feet regions using such as heating pads is a promising way to protect human feet. Compared to traditional thick and bulky sleeping bags, smart heating sleeping bags are slimmer and cheaper. Research findings presented in this study have evidently demonstrated that a smart heating sleeping bag is effective in improving wearers’ thermal comfort during sleeping under cold outdoor environments. Particularly in the camping sites and other outdoor recreation facilities where electricity can be easily accessed, using heating sleeping bags could be served as one of the best choices to provide wearers a better sleeping environment. For the outdoor settings where electric power is not available, a light and portable high capacity lithium-ion battery could be used to supply power to smart heating sleeping bags. However, it should be noted that such a high capacity battery, depends on its actual capacity, may only provide users a limited heating period (ranging from several hours to a number of days). Finally, the application of such smart heating sleeping bags to those settings as long-period hiking in the wilderness is greatly restricted due to the battery capacity and load concerns.

Limitations should be acknowledged that the weight increment in the smart sleeping bag is 464 g, accounting for about 45% in comparison to the weight of the traditional sleeping bag. This added load may lower the users’ enthusiasm to use the smart sleeping bags. The durations of the tests were three hours, somewhat shorter to mimic the normally use of the sleeping bags. In future studies, lighter smart sleeping bags would be designed and prolonged testing duration will be adopted to investigate their effectiveness in improving the wears’ local thermal comfort in the feet.

## Additional Information

**How to cite this article**: Song, W. F. *et al.* Use of a novel smart heating sleeping bag to improve wearers’ local thermal comfort in the feet. *Sci. Rep.* **6**, 19326; doi: 10.1038/srep19326 (2016).

## Figures and Tables

**Table 1:** Characteristics of the sleeping bag tested.

Sleeping bag	Composition	Weight (g)	*T*_*com-l*_ (°C)	*T*_*lim-l*_ (°C)	*T*_*com-c*_(°C)	*T*_*lim-c*_ (°C)
MAR_CON_	Shell: nylon; lining: polyester; filling: goose down; two heating pads at the feet region without heating power input.	1025	−2.4	−8.7	−0.4	−6.4
MAR_HT_	Shell: nylon; lining: polyester; filling: goose down; two heating pads at the feet region with 20 W heating power.	1489	–	–	−1.2	−7.2

−*not applicable.*

**Table 2:**
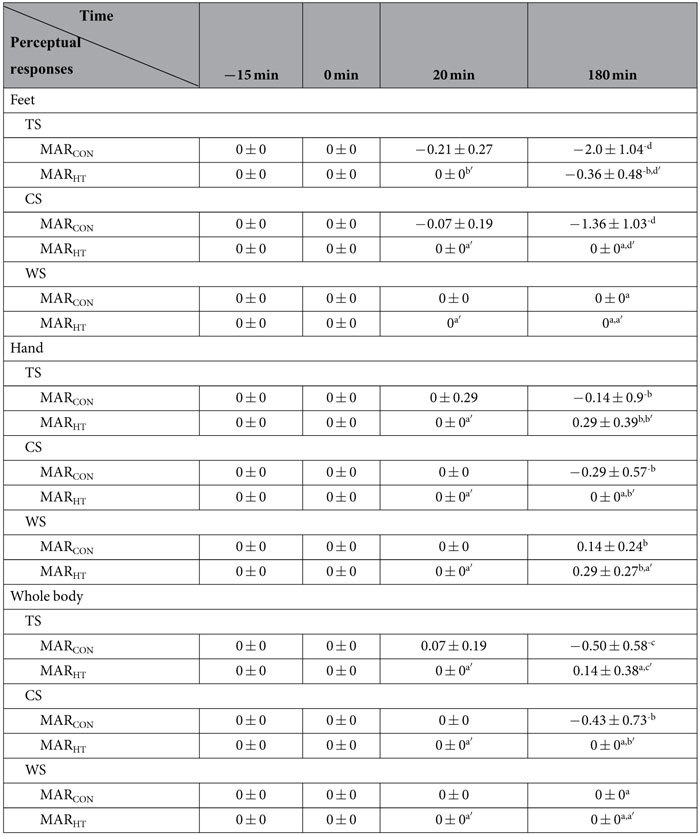
Perceptual sensations of the seven female subjects in the two sleeping bag test scenarios.

Difference in perceptual sensations between the time points at the 20^th^ min and the 180^th^ min of the exposure were presented as: 0–0.19^a^ = negligible effect, 0.20–0.49^b^ = small effect, 0.50–0.79^c^ = moderate effect and more than 0.8^d^ = large effect; Difference in perceptual sensations between MAR_HT_ and MAR_CON_ were expressed as: 0–0.19^a′^ = negligible effect, 0.20–0.49^b′^ = small effect, 0.50–0.79^c′^ = moderate effect and more than 0.8^d′^ = large effect.

**Table 3:**
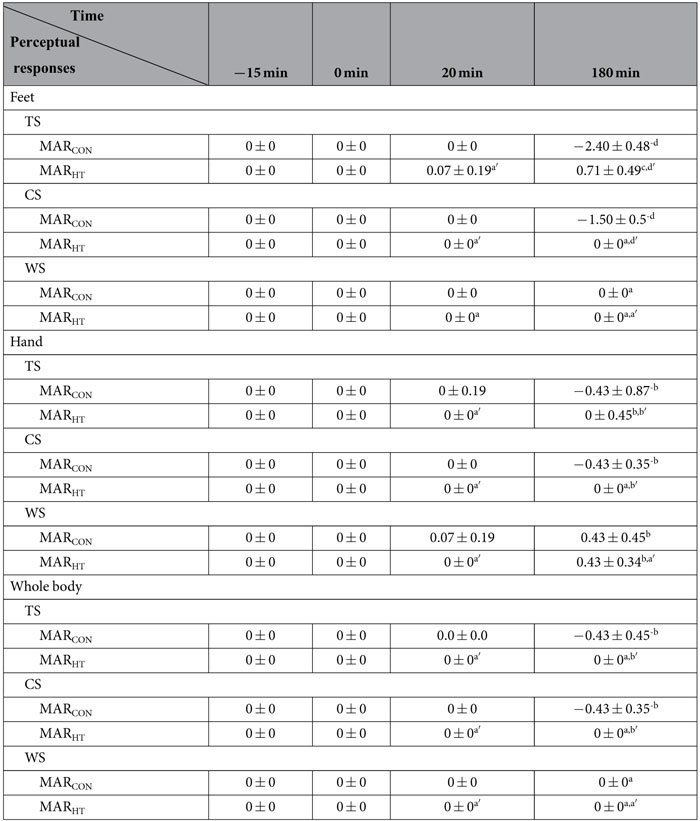
Perceptual sensations of the male subjects in the two sleeping bag test scenarios.
